# Variation of Biophysical Parameters of the Skin with Age, Gender, and Body Region

**DOI:** 10.1100/2012/386936

**Published:** 2012-03-12

**Authors:** Alireza Firooz, Bardia Sadr, Shahab Babakoohi, Maryam Sarraf-Yazdy, Ferial Fanian, Ali Kazerouni-Timsar, Mansour Nassiri-Kashani, Mohammad Mehdi Naghizadeh, Yahya Dowlati

**Affiliations:** ^1^Center for Research and Training in Skin Diseases and Leprosy, Tehran University of Medical Sciences, 415 Taleghani Avenue, Tehran 14166 13675, Iran; ^2^Fasa University of Medical Sciences, Fasa, Iran

## Abstract

*Background*. Understanding the physiological, chemical, and biophysical characteristics of the skin helps us to arrange a proper approach to the management of skin diseases. *Objective*. The aim of this study was to measure 6 biophysical characteristics of normal skin (sebum content, hydration, transepidermal water loss (TEWL), erythema index, melanin index, and elasticity) in a normal population and assess the effect of sex, age, and body location on them. *Methods*. Fifty healthy volunteers in 5 age groups (5 males and females in each) were enrolled in this study. A multifunctional skin physiology monitor (Courage & Khazaka electronic GmbH, Germany) was used to measure skin sebum content, hydration, TEWL, erythema index, melanin index, and elasticity in 8 different locations of the body. *Results*. There were significant differences between the hydration, melanin index, and elasticity of different age groups. Regarding the locations, forehead had the highest melanin index, where as palm had the lowest value. The mean values of erythema index and melanin index and TEWL were significantly higher in males and anatomic location was a significant independent factor for all of 6 measured parameters. *Conclusion*. Several biophysical properties of the skin vary among different gender, age groups, and body locations.

## 1. Introduction

The skin is the largest multifunctional organ in the body. It functions as a protective physical barrier by absorbing UV radiation, preventing microorganism invasion and chemical penetration, and controlling the passage of water and electrolytes. The skin has a major role in thermoregulation of body, in addition to immunological, sensory, and autonomic functions [1]. Understanding the physiological, chemical, and biophysical characteristics of the skin helps us to arrange a proper approach to the management of skin diseases. However, it is critical to consider the influence of genetic and environmental factors on most of the skin characteristics.

Man et al. assessed the differences in the skin surface pH, sebum content, and stratum corneum (SC) hydration at various ages and in both genders in a large Chinese population without skin diseases and concluded that these parameters vary with age, gender, and body site [2]. Marrakchi and Maibach established a preliminary map of the human face for 6 biophysical parameters in 9 locations and compared these various characteristics in different age groups [3]. 

 The aim of this study is to assess the biophysical characteristics of normal skin with standardized experimental conditions in an Iranian population in order to compare with other studies.

## 2. Materials and Methods

### 2.1. Volunteers

Fifty healthy volunteers in 5 age groups were examined: 10–20, 20–30, 30–40, 40–50, and 50–60 years old. There were 10 subjects in each group (5 females and 5 males). This study was approved by the ethics committee of Center for Research & Training in Skin Diseases & Leprosy and was performed according to the Declaration of Helsinki principles. All of the participants were instructed about the study and an informed consent was obtained from each one.

### 2.2. Methods

Eight body regions (forehead, cheek, nasolabial fold, neck, forearm, dorsal side of the hand, palm, and leg) were studied on their right sides. No skin care products were applied to the measured sites for at least 2 hours prior to the measurements. A small area of each location was wiped with ethanol 1 hour before the parameters were measured in a room at a temperature of 20–25°C and relative humidity of 30–40%.

Skin sebum content, hydration, TEWL, erythema index, melanin index, and elasticity were measured with respective probes Sebumeter, Corneometer, TEWAmeter, Mexameter, and Cutometer (Courage & Khazaka electronic GmbH, Cologne, Germany). Sebumeter SM 815 uses the difference of light intensity through a plastic strip to indicate the amount of absorbed sebum. The sebum level is expressed in *μ*g/cm^2 ^[4]. Corneometer CM 825 uses the high dielectric constant of water for analyzing the water-related changes in the electrical capacitance of the skin. It displays hydration measurements in system-specific arbitrary units [5]. A melanin index is calculated by Mexameter MX 18 from the strength of the absorbed and the reflected light at, respectively, 660 and 880 nm. An erythema index is processed similarly at, respectively, 568 and 660 nm [6]. The measurement of TEWL by TEWAmeter TM 300 is based on the diffusion in an open chamber and is measured as g/m^2^/h [7]. Cutometer MPA 580 pulls the targeted skin into the probe with a controlled vacuum pressure. Then the vertical deformation of the skin is measured and analyzed by computer softwares and is expressed arbitrarily [8]. 

### 2.3. Statistical Analysis

The data were analyzed with SPSS-16 software (SPSS Inc. Chicago Ill). A mixed model ANOVA was used for comparison of data between study groups. In this analysis age (in five levels) and sex were defined as the fix effect factors. A variable which contained subject codes was defined as random effect factor. Also locations of the measurement (8 locations) were defined as repeated factors. To specify the relationship between the levels of random effects (8 locations), an unstructured covariance matrix was chosen. *P* values <0.05 were considered significant.

## 3. Results

The mean and standard deviation of skin hydration, TEWL, melanin index, erythema index, sebum, and elasticity in both genders are shown in Table 1. Sex had an independent effect on TEWL, skin melanin index, and erythema index, but not on skin hydration, elasticity, or sebum.

The mean and standard deviation of these biophysical parameters in different age groups and body locations are shown in Tables 2 and 3, respectively. Age had a significant influence on skin hydration and melanin index (*P* < 0.05) and a marginally significant effect on elasticity (*P* = 0.05). Anatomic location was a significant independent factor for all of 6 measured parameters.

## 4. Discussion

### 4.1. Hydration

Stratum corneum hydration has an important role in skin functions such as regulating epidermal proliferation, differentiation, and inflammation [2]. In this study skin hydration was higher in female subjects, but the difference was not statistically significant (Table 1). Ehlers et al. [9] reported that the skin of females and males was hydrated equally. No correlation was found between skin hydration and sex in another study [10]. 

As reported by Man et al. [2], we detected a significant relationship between skin hydration and age (Table 2). Marrakchi and Maibach [3] reported that the oldest individuals had the least hydrated skin. One of the factors causing reduced stratum corneum hydration in the older group is a decrease in natural moisturizers [2]. In a study about the effects of menopause on physiological characteristics of the skin, late menopausal women had higher skin hydration than peri/premenopausal women [11]. However, some other investigations found no relation between skin hydration and age [10, 12, 13].

In concordance with our study, Shriner and Maibach [14] and also Marrakchi and Maibach [3] found out that neck had the most hydrated skin compared to the other parts of the face. This was due to high frequency conductance values of the neck [15]. Regarding ethnicity, it was reported that hydration of the skin and also the effect of age on hydration were influenced by ethnicity [16, 17]. However, in other studies skin hydration showed no significant difference among ethnicities [18, 19]. Some of the dissimilarities between this study and others can be explained due to ethnical and environmental variations.

### 4.2. TEWL

Transepidermal water loss is used to assess skin water barrier function. We found out that TEWL was higher in males than that in females (Table 1). Males usually have more outdoor activities and their skins are more damaged. This is in contrast to the studies done by Ehlers et al. [9] who reported equal TEWL in both sexes. However, another research found no relation between TEWL and sex [10]. 

We found that TEWL was lower in the youngest and in the oldest subjects, but age did not show a significant effect on TEWL. A negative correlation between age and TEWL has been reported in several studies [10, 20–22]. However, Marrakchi and Maibach found no correlation between these two parameters [3]. Also, Shriner and Maibach found no relation between TEWL and perceived age [14]. 

In this study, the palm and the leg had the highest and the lowest TEWL, respectively (Table 3). Palm is believed to be an exception. Despite the great thickness of the stratum corneum of the palm, it is the low amount of stratum corneum barrier lipids which causes the high level of TEWL on palm [15]. Marrakchi and Maibach [3] reported that TEWL was significantly higher in the nasolabial fold than the forehead. Tagami [15] showed that TEWL of forehead and the nasolabial fold were significantly higher than the cheek. On the other hand, Lopez et al. [22] and also Le Fur et al. [23] reported that TEWL of the cheek was significantly higher than that of the forehead. Variations in TEWL levels are due to different factors such as skin blood flow, skin temperature, the stratum corneum lipid contents, and the degree of corneocyte formation [3]. Moreover, our sample size, ethnicity, and methodological differences may have affected the results. Wesley and Maibach reported that TEWL was greater in black skin compared with white skin, but it was inconclusive in Asians [19]. Another study showed no difference in TEWL between Black, African, or Carribean Mixed-race and Caucaisan women [18]. 

### 4.3. Sebum

In this study, sex did not have a significant effect on sebum, although skin sebum content was higher in males (Table 1). It is known that sebum production correlates positively with testosterone levels in both sexes, through dehydroepiandrosterone in males and etiocholanolone in females [2]. Other studies also have shown that sebum levels were the same in both sexes [9, 10]. 

We did not find significant difference in skin sebum content among age groups (Table 2). Additionally, another study found no relation between skin sebum and age [10]. In a report from Switzerland [12], skin sebum level decreased with age. Furthermore, Ohta et al. [11] reported that skin sebum content is reduced after menopause in women. The differences may be due to sample size and ethnicity.

We found out that sebum secretion was the highest on the nasolabial fold and the lowest on the leg (Table 3). Also Lopez et al. [22] and Tagami [15] reported that skin sebum level was significantly higher in the forehead than that in the cheek. Another study found out that sebum level was the highest in the central areas of the face such as the nasolabial fold in young individuals. Some factors such as hormones, age, sex, and ethnicity could affect the sebum secretion; therefore, standardized experimental methods and conditions are required [3]. Castelo-Branco et al. Maibach reported that lipid contents were different in regarding ethnicity, but they were inconclusive [24]. In another study, the effects of ethnicity on skin lipid content were assessed but no significance was reached [19]. 

### 4.4. Skin Pigmentation

Melanin is one of the pigments which determine the skin color [25]. In our study, skin melanin index was significantly higher in males (Table 1). We also found out that subjects aged 20–30 years and 10–20 years had the highest and the lowest skin melanin index, respectively (Table 2). However, in a study which was done in China [13], no correlation was found between skin pigmentation and age. In our study, forehead was the most pigmented area, whereas the palm had the lowest skin melanin index (Table 3). This can be explained by the degree of sun exposure. A study which was conducted in Japan reported that individuals who lived in sun-exposed areas had higher skin melanin index compared to people who lived in less sun-exposed areas [26]. Hermanns et al. found out that the pattern of melanin index variation in different body parts was irrespective of the skin phototype and the dorsal forearm always had the highest melanin index [27]. 

### 4.5. Erythema Index

Quantification of erythema and melanin is useful for analysis of skin tests and management of skin diseases [6]. Personal factors (age, sex, race, anatomical site, skin surface properties), environmental factors (light conditions, temperature), and different procedures influence skin colour [28]. We found out that skin erythema index was higher in males than females (Table 1) but was not significantly different among age groups (Table 2). Regarding body location, the nasolabial fold had the highest erythema index. On the other hand, leg had the lowest skin erythema index (Table 3). In a study done in Belgium, 4 parts of the body were investigated in 137 normal individuals. They found out that the forehead had the maximum erythema index. Also they concluded that regional variability in erythema index was unpredictable [27]. Clarys et al. compared three skin color measurement instruments (Chromameter, Dermaspectrephotometer, and Mexameter) by evaluating several parameters such as erythema index in normal individuals. They found out that Chromameter was capable of measuring all colors, while the reflectance meters (Mexameter and DermaSpectrometer) were suitable for evaluating the intensity of erythema and melanin-induced pigmentation [25]. 

### 4.6. Elasticity

Skin elasticity was higher in female subjects than in males (Table 1); however, the difference was not statistically significant. Also Ishikawa et al. [29] reported that skin elastic properties were not correlated with sex. On the other hand, the oldest age group had the least skin elasticity (Table 2), which is in concordance with another study done by Wendling and Dell'Acqua [12] The highest skin elasticity content was observed in the age group of 20–30 years. It was reported that skin collagen content showed a peak between the ages of 20 and 40 years and decreased between the ages of 40 and 60 years [24]. Some studies [29–31] found a negative correlation between forearm skin elastic properties and age in women. Sumino et al. [31] reported that skin elasticity decreased after menopause 0.55% per year; however, it increased by 5.2% after 12 months of hormone replacement therapy. It is known that severe disorganization of the elastic fiber network and decrease in the collagen fiber bundles occur with age. We found out that the neck and the leg had the most and the least skin elasticities, respectively (Table 3). In another study in which 4 parts of the body were examined (finger, hand, forearm, and chest), it was reported that skin elastic property of the chest was the highest [29]. These differences are mainly due to alterations in the elastic fiber network.

## 5. Conclusion

In this study we showed variations in several biophysical properties of the skin among different gender, age groups, and skin locations. These differences may be involved in the individual susceptibility to skin diseases. On the other hand, they should be considered in the formulation of skin care products. Genetic and environmental factors, methodology, and sample size might be involved in the variations in biophysical properties of skin reported in various studies.

## Figures and Tables

**Table 1:** The mean and standard deviation of skin hydration, TEWL, melanin index, erythema index, elasticity, and sebum according to gender.

Variable	Male	Female
Hydration	48.42 ± 22.12	49.06 ± 16.09
TEWL	15.49 ± 11.47	9.52 ± 7.36
Erythema index	378.14 ± 124.50	303.63 ± 100.73
Melanin index	214.82 ± 77.66	176.82 ± 58.42
Elasticity	0.270 ± 0.142	0.273 ± 0.121
Sebum	60.39 ± 74.52	42.19 ± 54.10

**Table 2:** The mean and standard deviation of skin hydration, TEWL, melanin index, erythema index, sebum, and elasticity in 5 age groups.

	10–20	20–30	30–40	40–50	50–60
Hydration	49.74 ± 19.25	47.08 ± 16.61	50.53 ± 17.69	53.34 ± 20.78	43.04 ± 20.58
TEWL	9.18 ± 6.46	14.90 ± 12.59	13.67 ± 8.99	14.64 ± 11.08	9.87 ± 8.50
Melanin	174.25 ± 58.55	235.95 ± 82.15	210.14 ± 76.04	181.10 ± 57.90	179.51 ± 63.68
Erythema	323.25 ± 125.42	370.36 ± 113.74	336.22 ± 122.64	337.62 ± 113.42	328.32 ± 117.91
Sebum	53.75 ± 77.94	50.10 ± 51.81	42.06 ± 60.42	66.71 ± 73.42	41.77 ± 57.72
Elasticity	.2561 ± .1118	.3025 ± .1566	.2887 ± .1228	.2803 ± .1211	.2345 ± .1348

**Table 3:** The mean and standard deviation of skin hydration, TEWL, melanin index, erythema index, sebum, and elasticity according to body location.

	Forehead	Cheek	Nasolabial fold	Neck	Forearm	Dorsal	Palm	Leg
Hydration	53.54 ± 16.49	62.12 ± 15.63	38.19 ± 18.02	62.88 ± 15.28	51.00 ± 15.92	44.14 ± 17.43	40.47 ± 18.47	37.22 ± 17.50
TEWL	12.27 ± 10.05	9.57 ± 7.22	14.05 ± 8.25	10.47 ± 9.23	10.12 ± 9.54	9.86 ± 8.84	23.47 ± 9.67	9.68 ± 9.52
Melanin	228.16 ± 66.48	203.35 ± 50.53	202.29 ± 54.01	225.96 ± 66.41	193.31 ± 70.34	225.94 ± 67.25	98.98 ± 41.75	189.71 ± 62.31
Erythema	420.49 ± 90.31	399.80 ± 91.12	480.42 ± 88.93	373.27 ± 97.31	257.88 ± 69.87	331.94 ± 62.23	248 ± 60.82	205.00 ± 63.52
Sebum	95.65 ± 51.38	73.39 ± 64.05	136.98 ± 72.33	64.41 ± 64.51	18.45 ± 37.88	8.84 ± 8.45	9.82 ± 10.11	2.88 ± 6.42
Elasticity	.2901 ± .1072	.3040 ± .0820	.3057 ± .1051	.4528 ± .1102	.2783 ± .0775	.2280 ± .0851	.1819 ± .1339	.1373 ± .0685
